# Co-creation of a patient engagement strategy in cancer research funding

**DOI:** 10.1186/s40900-023-00501-x

**Published:** 2023-09-29

**Authors:** Michael S. Taccone, Nathalie Baudais, Don Wood, Suzanne Bays, Sasha Frost, Robin Urquhart, Ian D. Graham, Judit Takacs

**Affiliations:** 1 Childhood Cancer Survivor Canada, Toronto, ON Canada; 2 Canadian Cancer Society, 55 St Clair Ave W Suite 500, Toronto, ON M4V 2Y7 Canada; 3 Department of Community Health and Epidemiology, Dalhousie University, Halifax, NS Canada; 4 Schools of Epidemiology and Public Health & Nursing, University of Ottawa, 600 Peter Morand Crescent, Ottawa, ON K1G 5Z3 Canada; 5 Ottawa Hospital Research Institute, 501 Smyth Road, Box 241, Ottawa, ON K1H 8L6 Canada

**Keywords:** Patient engagement, Research funding, Strategy development, Co-creation

## Abstract

**Background:**

As research teams, networks, and institutes, and health, medical, and scientific communities begin to build consensus on the benefits of patient engagement in cancer research, research funders are increasingly looking to meaningfully incorporate patient partnership within funding processes and research requirements. The Canadian Cancer Society (CCS), the largest non-profit cancer research funder in Canada, set out to co-create a patient engagement in cancer research strategy with patients, survivors, caregivers and researchers. The goal of this strategy was to meaningfully and systematically engage with patients in research funding and research activities.

**Methods:**

A team of four patient partners with diverse cancer and personal experiences, and two researchers at different career stages agreed to participate as members of the strategy team. Ten staff members participated in supportive roles and to give context regarding different departments of CCS. The strategy was co-developed in 2021/2022 over a series of 7 workshops using facilitation strategies such as ground rules and consensus building, and methods such as Design Thinking. The strategy was subjected to 3 rounds of validation.

**Results:**

The co-creation and validation process resulted in a multi-faceted strategy with actionable sections, including vision, guiding principles, engagement methods, 13 prioritized engagement activities spanning the spectrum of research funding, and an evaluation framework. The experience of co-creating the strategy was captured using the Patient and Public Engagement Evaluation Tool and revealed a positive, supportive experience.

**Conclusions:**

Lessons learned included the value of an emphasis on a co-creation process from day one, the utility of facilitation techniques such as ground rules for dialogue, consensus building and Design Thinking, and the importance (and challenge) of designing for and incorporating equity when drafting the strategy. Future work will include implementation and evaluation of the strategy, as well as an examination of further ways to meaningfully and systematically engage diverse voices in research and research funding.

**Supplementary Information:**

The online version contains supplementary material available at 10.1186/s40900-023-00501-x.

## Abstract (plain language summary)

As researchers and healthcare providers see benefits of patient engagement in cancer research, research funders are also looking to engage with patients in their funding processes and research activities. The Canadian Cancer Society (CCS), the largest non-profit cancer research funder in Canada, set out to co-create a patient engagement in cancer research strategy with patients, survivors, caregivers and researchers. The goal of this strategy was to meaningfully and systematically engage with patients in research funding and research activities. Four patient partners and two researchers were supported by ten CCS staff members to co-create the strategy in 2021/2022 over a series of 7 workshops. They used facilitation strategies such as ground rules and consensus building, and methods such as Design Thinking. The strategy was then validated. Co-creation resulted in an easy-to-use strategy with actionable sections, including vision, guiding principles, engagement methods, 13 prioritized activities, and an evaluation framework. The experience of co-creating the strategy was captured using a well-regarded evaluation tool and revealed a positive, supportive experience.    Lessons learned during the process included making sure the co-creation process started on day one, the usefulness of facilitating the process, and the importance of considering issues of equity when drafting the strategy.

**Supplementary Information:**

The online version contains supplementary material available at 10.1186/s40900-023-00501-x.

## Background

Patient engagement in health research is fast becoming a usual practice in North America as well as many countries in Europe and Australia [1–5]. The term *patient engagement* in our context refers to the inclusion of patients in research activities or in the funding process of research as contributors and/or decision makers. As members of research teams, granting committees, clinical trial oversight committees or other levels of planning or implementing human research, patients are included to contribute their lived and living-experience, perspective, and knowledge to help shape research with the intention of improving its relevance, impact, and eventual translation to the patient populations it serves [6, 7].

Patient engagement has become increasingly integrated into funding models and more recently embedded within funding organizations themselves [1, 8]. Some countries have begun to outline best practices of patient engagement in research, including the roles of both government and non-government research funders, largely due to patient advocacy efforts at lobbying government and policy makers, as well as consensus among medical and scientific communities that patient engagement leads to improvements across the sector [7, 9, 10]. Furthermore, non-profit and international organizations have also established models, guidelines, and training programs for professionals, researchers and patients in patient engagement [10, 11]. With patient engagement now being supported at the government level and at the research level, it has become imperative that funding organizations accordingly adapt to establish strategies to include patients and caregivers in their engagement frameworks.

In Canada, most health research is financially supported by federally-funded health agencies such as the Canadian Institutes of Health Research (CIHR) or by national or provincial disease-based charities. Of all disease specific research in Canada, cancer research receives one of the highest amounts of funding. Given that 2 in 5 people in Canada are anticipated to be diagnosed with cancer in their lifetime, cancer research is one of the most active and productive sectors of health research in the country. Among cancer foundations and charities, the Canadian Cancer Society (CCS) is the largest national charitable funder of cancer research in the country. Since 1947, the organization has invested over $2 billion in cancer research [12], and currently invests more than $40 million per year. In 2021, in response to the growing importance of patient engagement including the evidence supporting patient engagement in research funding from the Patient-Centered Outcomes Research Institute [13], CCS sought to establish its own patient engagement strategy [14]. What followed was, to the authors’ knowledge, the establishment of the first patient engagement strategy in a major non-governmental cancer research funding organization. The experience of CCS in the establishment of a multi-faceted patient-engagement research strategy and how this approach can be used and adapted to similar organizations globally are outlined in this paper.

## Methods

### Objectives

The objectives of this project were to: (1) co-create a patient engagement strategy in cancer research funding for the Canadian Cancer Society (CCS), (2) describe the tools and approaches used in co-creation, and (3) describe successes and lessons learned.

CCS is Canada’s largest national charitable funder of cancer research and supports all Canadians living with cancer across the country. The organization’s mission is, in trusted partnership with donors and volunteers, to improve the lives of all those affected by cancer through world-class research, transformative advocacy, and compassionate support. In 2021, CCS invested $44.3 million in research to help prevent cancer, enhance screening, diagnosis and treatment, and ensure people diagnosed with cancer can live longer, fuller lives. As part of the organization’s research strategy, several integrated approaches were identified as essential to success. Meaningful patient partnership within research is one of these integrated approaches. Thus, in 2021, CCS set out to co-create a strategy for engaging patients in research funding, specific to the CCS context. A Strategy Development Team (the Team) was formed to co-create the strategy with patients and researchers from the beginning.

### Strategy development team

Four patient partners with varying levels of patient partnering experience, cancer experience and in different geographical locations were invited and agreed to participate as members of the Team. These individuals were selected because of their previous experience in patient engagement and strategy development and varied cancer experiences, gender, age, and location. Our collective group experience included caregiving for multiple individuals, multiple cancer types, some medical training and PhD candidacy, as well as experience with business, leadership training, board experience, and cancer charity experience. We acknowledge that people are complex with multiple identities and that one identity does not take away from any other identity they may hold. We viewed these experiences as a benefit in that Team members could provide additional perspectives; however all members were very clear to describe what perspective they were providing input from and for patient and caregiver partners they were asked to first consider issues from that perspective. All patient and caregiver partners were chosen with some prior experience and exposure to research as patient/caregiver partners. Recruitment was by word of mouth and was open to patients and caregivers. CCS staff across the organization were asked to provide suggestions for patient partners currently or previously engaged with CCS or for connections with community or patient organizations where this opportunity could be shared. With some aspects of diversity in mind (cancer experience and role, geography) and with a goal of recruiting patient partners with experience with cancer research and strategy development, a shortlist of potential patient partners was created. Patient partners were sent individual invitations describing the strategy development opportunity. The first four patient partners that were invited accepted, and with the goal of a small team size for agility, including researchers and staff, researchers were sought next in a similar fashion. For researchers, we prioritized experience with patient engagement and diversity in location and career stage. Two researchers representing early and later career stages were invited and also participated as members of the Team. A formal invitation letter was provided to all participants external to CCS. The demographics for the Team are described in Table 1. CCS staff members from multiple departments and areas participated primarily in supportive roles and to give context regarding CCS, with staff members encouraged to self-select sections of the strategy of interest or expertise to limit staff representation and group size. A total of ten CCS staff members supported the Team at different times during the development process.

**Table 1:** Demographics

	Core strategy development team	Validation—key informants	Validation—survey	Validation—advisory council
Total N	6	8	166	13
Gender (man/woman/non-binary/two-spirit/prefer not to say^+^)	3/3/0/0/0	1/7/0/0/0	46/115/0/0/2	8/5/0/0/0
Cancer experience (patient/caregiver)	2/2	7/1	147/11	0/1
Researcher* (n)	2	2	3	13
Underserved community representation** (n)	Rural and remote (1), young adult (1)	Black (2), South Asian (1), East Asian (1), francophone (1), rural and remote (2), young adult (1)	Black and racialized groups (25), francophone (27), rural and remote (50), young adults (10)	East Asian (1)

^+^The question on gender also included the option to self-describe. No respondents chose this option

^*^Researcher denotes an individual that conducts research as part of their daily work or career

^**^Young adult is defined as an individual who was diagnosed with cancer between ages 15 and 39

Some survey respondents opted not to disclose gender or other identity information (demographic questions were optional)

### Strategy co-creation process

**Fig. 1:**
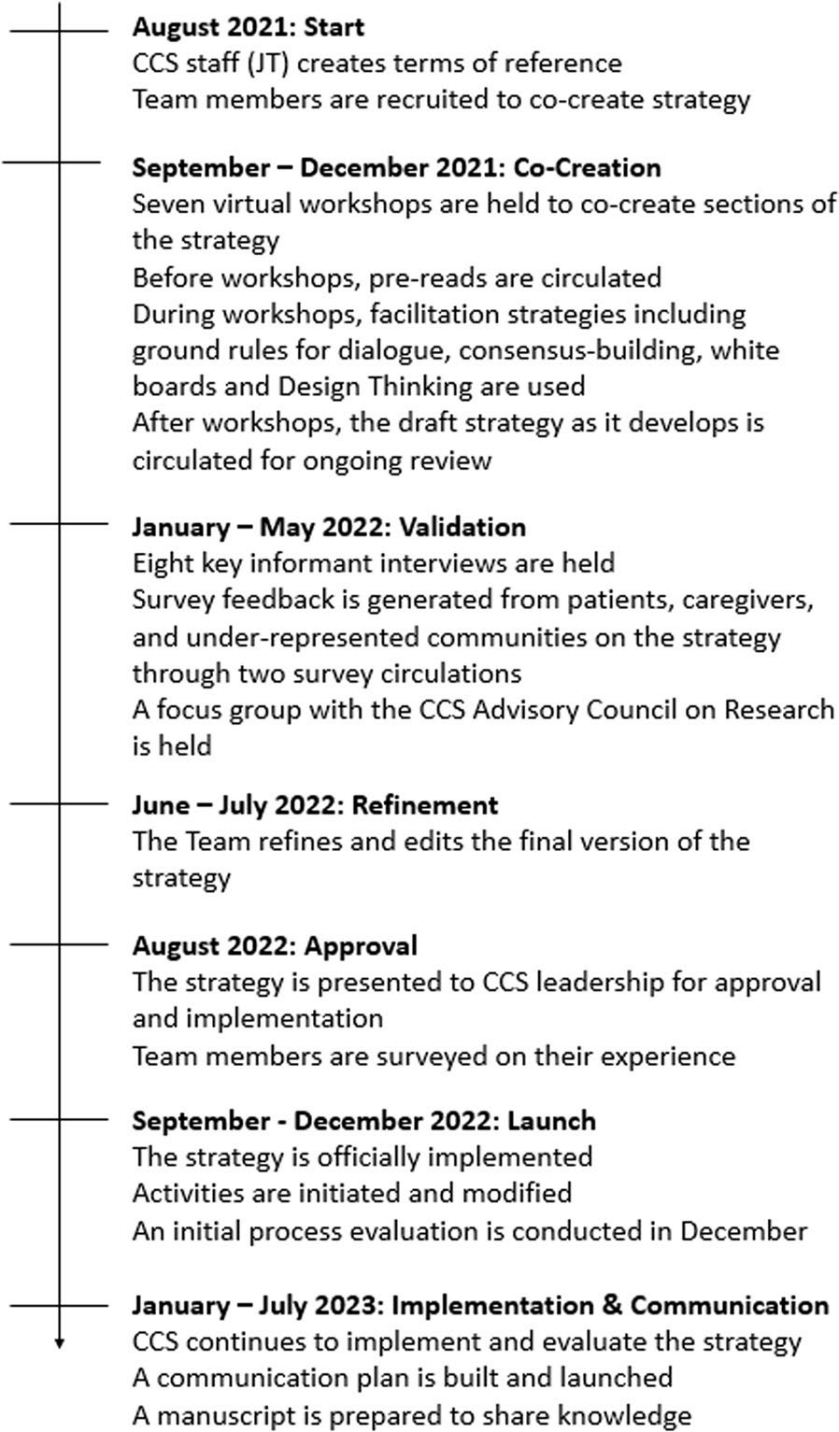
Timeline of strategy co-creation

The timeline and steps of co-creating the strategy are described in Fig. 1. With the goal of patient co-creation of the strategy from the outset, the CCS staff lead (JT) drafted a terms of reference document that described expectations of each Team member in strategy co-creation including roles and responsibilities, decision-making processes, estimated compensation, expectations of confidentiality and code of conduct, anticipated work, and evaluation processes. Also included in the terms of reference was an estimate of the time commitment including anticipated workshops, meetings and consultation for each section of the strategy. All patient partners, researchers, and staff involved in strategy creation were provided with the terms of reference. This document was used to discuss strategy creation with the recruited Team members. Patient partners were engaged from the very beginning in co-creating the patient engagement strategy to harness their expertise on involving patients in research funding processes emulating the motto “nothing about us without us”. The group size for creation of each section of the strategy was set at 12–15 members for optimal dynamics in co-creation.

The Team set out to establish the following strategy sections through a series of virtual workshops: guiding principles, core areas for engagement, levels (spectrum) of engagement, proposed patient engagement activities, impact and evaluation. Building the strategy was dynamic, and extra workshops were added where needed (for example, two workshops each for developing patient engagement activities and evaluation, and a workshop to review the draft strategy and prioritize the activities was added). The final list of workshops was:

- Guiding Principles
- Core Areas for Engagement and Levels of Engagement
- Proposed Patient Engagement Activities (2 workshops)
- Impact and Evaluation (2 workshops)
- Strategy Review and Prioritization

Workshops were 90 min to two hours long and were held online using MS Teams or Zoom between September and December 2021. Facilitation strategies including ground rules for dialogue, consensus-building (Fist-to-Five Agreement Method) [15] and white boards for co-working were used, as well as Design Thinking [16] in the development of proposed activities. Ground rules for dialogue consists of a set of statements on conduct that the group agrees to at the beginning of an activity. The Fist-to-Five Agreement Method is a quick version of consensus building used throughout the activity to check level of agreement with decisions and actions—from strong disagreement (fist) to complete agreement (palm out/five fingers out). If any Team members displayed two fingers or less at any time, a discussion would be held to understand the concerns and take any actions necessary as a group. Design Thinking is an iterative process of generating and testing ideas as a group, and use of the technique is described below.

Prior to each workshop, an agenda and pre-reads were circulated (i.e., workshop methodology, review of work to date). A follow up email containing the draft strategy as it was created was circulated after each meeting to ensure what was heard during the meeting was reflected in the strategy and Team members could provide input in real time. All Team members had ongoing access to the strategy as it developed and continuously provided feedback. Aside from agreed upon workshop topics, remuneration and authorship (publications related to the work of the Team) were also discussed and undertaken by the Team via email after patient partners flagged the importance of discussing these topics early on.

### Strategy validation

After the initial draft of the strategy was completed, the Team sought feedback from the larger community, primarily, patients, caregivers and researchers for validation. There were three feedback mechanisms. First, specific advisors (key informants) were sought—patients with extensive strategy experience, patients with no previous exposure to CCS, and patients from underserved communities including those living in rural and remote areas, Black, South Asian and East Asian patients, francophone patients, and young adults diagnosed with cancer. Key informants who had previous contact with CCS and who had expressed interest from outreach to community and patient organizations were invited. The key informants were provided with the strategy for review and then 1:1 60-min interviews were conducted by the CCS staff lead (JT) with each advisor. Key informants were given the draft strategy to review and asked open-ended questions on each section to identify what was important and what could be improved. For example, the following questions and prompts were included in the qualitative interview guide regarding Guiding Principles: Do you think these principles are appropriate and should guide CCS’ patient involvement efforts? Do you think any of these principles should be changed or removed? Do you think there are any principles that are missing and should be added? Answers were transcribed and then collated into a master spreadsheet of all key informant feedback. The verbatim feedback was categorized under the section of the strategy it was referring to and any suggested change was extracted into a separate column for consideration by the Team. Duplicate entries were grouped together and statements where no change was suggested were coded as ‘N/A’ in the change column. For example, “[I’m] really excited about the idea to be part of the creation of something new” was coded as N/A, where “The Award for Excellence is nice to have, not critical” was coded as a change (reducing the priority level of this engagement activity) for the Team to consider.

Next, the draft strategy was presented to the CCS Advisory Council on Research for review and feedback during a focus group discussion in January 2022. The Advisory Council on Research consists of 13 researchers from various areas of cancer research, geographic locations and institution sizes who advise CCS on research funding processes and decisions. An abbreviated version of the strategy was shared as a pre-read report and presented in greater detail in a presentation. Open feedback and discussion were solicited during a short (20-min) discussion, with prompts such as “What challenges do you foresee? What should be changed or modified about this strategy?”. All feedback was captured by a notetaker, and then summarized by the strategy staff lead (JT). Potential modifications to the strategy were then added to a table of modifications for review by the Team.

Finally, to purposefully seek feedback on the strategy from individuals from racialized and underserved communities, CCS solicited input from a broad sample of patients and caregivers via CCS’ patient and caregiver community and external community and cancer organizations. The survey included a summary of the overarching aim of the strategy, proposed activities for patient engagement, guiding principles, core areas, and spectrum of engagement. The survey asked respondents to rate the importance of the proposed activities (very important, somewhat important, not important, not at all important, or unsure) and comment on what was missing or should be modified in each section. A question on whether the strategy would help CCS partner with patients meaningfully and systematically in research funding (the strategy aim) was also included (strongly agree, somewhat agree, disagree, strongly disagree or unsure). Finally, respondents were invited to provide any other feedback they wished about the strategy. The Team reviewed the survey before it was circulated. The survey was available for 4 weeks from February 2 to March 2, 2022. CCS generated a convenience sample of an internal patient and caregiver panel, CCS’ online patient and caregiver community, patient advocacy organizations, CCS staff and CCS-funded researchers, soliciting feedback on the survey with the goal of 30–50 responses estimated for qualitative data saturation [17]. Data were analyzed similarly to the validation rounds described above, with quantitative feedback on level of importance of each activity informing any changes in activity prioritization, and qualitative feedback categorized by strategy section and suggestions for change extracted into a table for review by the Team.

Feedback from all three approaches were collated and presented back to the Team for incorporation into the strategy in a virtual workshop in March 2022. Upon review of the responses to the survey, the Team noticed a lack of diversity in survey respondents and re-distributed the survey for 4 more weeks from April 15–May 15, 2022, with the goal of an additional 20 responses. The second wave of the survey was targeted at organizations representing underserved communities and identities such as black and racialized groups, those living in rural and remote areas, and young adults diagnosed with cancer. Contacts at organizations were asked to circulate the survey to their members and networks. Because the survey was sent to communities and organizations to circulate to their members, the exact number of people who were sent the first or second round of the survey is unknown. A second virtual workshop in June 2022 was scheduled to review the additional survey data received. The strategy was then refined with final edits made by the entire group.

### Experience of strategy co-creation

After completion of the patient engagement strategy, all team members involved in creating the strategy (including the 6 core members of the Team and eight CCS staff members—the two CCS staff survey designers did not participate) were surveyed using the Public and Patient Engagement Evaluation Tool (PPEET) [18]. This is a widely used tool designed to obtain a participant’s assessment of the engagement process [19] and is comprised of 5-point Likert-scale questions and open-ended questions grouped into four categories: Communication and Supports for Participation; Sharing your Views and Perspectives; Impacts and Influence of the Engagement Initiative; Final Thoughts. The PPEET has undergone assessment for and is considered to have good content validity [19] and reliability [20]. We summarized responses to discrete choice PPEET questions as frequencies. Because there was a small number of submissions, we did not conduct quantitative analyses. Free-form responses are reported as themes below.

The Guidance for Reporting Involvement of Patients and the Public (GRIPP2-LF) [21] was used to guide reporting of this paper and documents the level of patient engagement in the project. The GRIPP2-LF checklist is Additional file 1.

## Results

### Strategy co-creation

The strategy co-creation process began with the Team co-designing guiding principles. This involved the Team reviewing examples of guiding principles by other organizations such as the Canadian Institutes of Health Research Strategy for Patient-Oriented Research [22] and the McMaster University Collaborative for Health and Aging [23]. The Team then used the Tamarack Institute Creating Guiding Principles tool [24] to guide the creation of principles for the CCS research context. The Team initially identified seven guiding principles. The principles underwent several rounds of revision until agreement was reached. See Table 2 for the original and revised principles.

**Table 2:** Original and Revised Guiding Principles

Principle	Original version	Revised version
Safety	Principle not present in original version	We strive to create and support safe physical/virtual, emotional, and cultural spaces for respectful discussion and collaboration. This begins before the engagement occurs, during engagement, and continues even after engagement has ended
Mutual respect	CCS staff, patient partners, researchers, and all stakeholders acknowledge and value each other's expertise and experiential knowledge	We acknowledge and value each other's expertise and experiential knowledge, recognizing equitable power among collaborators
Co-build	CCS staff, patient partners, researchers, and all stakeholders work together from the beginning to identify problems and gaps, set priorities, co-create, evaluate, and implement solutions	We work together from the beginning to identify problems and gaps, set priorities, co-create, evaluate, and implement solutions
Diversity of experiences	CCS encourages multiple patient perspectives, prioritizing inclusivity, equity, and accessibility	We require multiple patient perspectives, prioritizing inclusivity, equity, and accessibility
Clear communication	Goals and expectations are outlined by project team members, touchpoints are set, and roles and responsibilities for all team members are defined	Goals and expectations are outlined by project team members, interpersonal boundaries and touchpoints are set, and roles and responsibilities for all team members are defined
Knowledge exchange	Information is shared in a context of trust between all stakeholders for equitable power, mutual understanding, education, and support	We share information in a context of trust and for mutual understanding, education, and support, while respecting confidentiality. We strive to recognize different forms of knowledge, bias and power dynamics through critical reflexive thinking
Personalized and progressive	Patient partner expertise is matched with projects, and both engagement and acknowledgment of engagement is tailored to the individual. Reflecting the nature of research, engagement will be meaningful and use a person-centered approach that evolves over time	Patient partner expertise is matched with projects, and patient partners are offered control and choice in engagement opportunities where possible. Both engagement and acknowledgment of engagement are tailored to the individual. Reflecting the nature of research, engagement will be meaningful and use a trauma-informed, person-centered approach that evolves over time, as needed
Accountability	All stakeholders demonstrate social responsibility and take ownership of the projects and decisions they are involved in	We demonstrate social responsibility and take ownership of the projects and decisions we are involved in

The Team developed the core areas and spectrum of engagement using a similar method, reviewing versions by the International Association for Public Participation [25] and the Canadian Medical Association [26] before co-creating the version for CCS. The Team proposed two core areas (governance & decision-making, capacity development) and 4 levels of engagement (informing, consulting, co-creating, leading). During the workshops, the Team reached consensus using the Fist-to-Five Agreement Method [15].

The Team then generated ideas for patient engagement activities to achieve progress in the core areas identified, using a modified version of Design Thinking [16] (Ideate and Prototype stages) and 10 × 10 idea generation [27]. One workshop was dedicated to generating ideas, where a total of 60 ideas were generated, with the second workshop focused on refining and prototyping ideas, as well as beginning prioritization of activities.

For impact and evaluation, the Team co-created conditions for success of patient engagement in research funding. Six conditions were developed in an initial thought storming and refined with example metrics in a second workshop. To the rest of the strategy, other sections were added in further rounds of revision, including a section acknowledging the importance of compensation for patient partners and an initial look at operational considerations for implementing the strategy.

A workshop was then held where the full strategy was reviewed and the top 12 proposed activities were prioritized (as ‘critical’, ‘high priority’ and ‘important’ in achieving the aim of partnering in meaningful and systematic ways with patients in CCS’ cancer research funding processes) for recommendation to leadership. The list of proposed patient engagement activities in CCS research funding can be found in Table 3 (one was added during validation for a total of 13). The Team acknowledged that while all ideas had merit and ideally should be pursued, the aim would be to resource and implement those activities under the Critical category within one year, with High Priority and Important activities pursued if resources allowed. It was also acknowledged that some activities were labelled Important because they were operationally more complex to complete and needed more time or more background work before being implemented, and that this was not only a reflection of the importance of the activity in successfully reaching the overall aim of the patient engagement strategy. Consensus on prioritization was sought using Fist-to-Five Agreement. Also at this workshop, the entire strategy was reviewed and the Team reached consensus on the strategy sections and content. CCS encouraged Team members throughout the strategy co-creation process to review progress to date and sections of the report for accuracy and completeness.

**Table 3:** Proposed patient engagement activities in CCS research funding

Critical activities	For example…
Engage patients with diverse perspectives in co-creation and leadership roles, to shift decision-making to more equal representation	Support informed participation of patients on research advisory councils
Include patients as review panel members, giving equal weight for different types of reviewer expertise	Expand and improve CCS’ Patient/Survivor/Caregiver Reviewer Program
Integrate patient engagement into research competitions (review and funding) broadly	Co-develop funding opportunities with patients
Have patients co-develop stories to share the significance of research work	Support patient leadership in telling their stories about working collaboratively with researchers
Engage patients in communicating this strategy	Co-communicate the shift in culture and practice with patients
Connect the needs of researchers with the skills and interests of patient partners	Create an ongoing program for patient-researcher matching, beginning with a pilot

High priority activities	For example…
Offer trauma-informed engagement resources and training	Collate and create (or use external) trauma-informed engagement training and resources for CCS staff and researchers
Engage patients in the identification of research priorities for strategic investment	Consider piloting patient-researcher research prioritization or creating targeted funding opportunities to do so
Design training for patients and researchers on patient engagement	Consider a workshop format for patients and researchers to collaborate and learn together about working together

Important activities	For example…
Establish an Award for Excellence focused on patient engagement	Focus on highlighting and promoting best practice exemplars, chosen by a team of patient reviewers
Engage patients in supporting the life cycle of research—development and design through to dissemination of research projects	Require the incorporation of patient partners, patient and caregiver engagement throughout research proposals for CCS research funding
Engage patients in review of research progress using updates	Update patients on grants that include patient partners or patient engagement
Engage patients in co-creating manuscripts and contributing to authorship	Consider creating a policy for CCS funded projects to include patients as co-authors

### Strategy validation

Validation (confirmation or modification of the strategy sections based on larger group input) was undertaken by collecting feedback on the completed draft strategy. Eight key informant interviews were conducted where notes were transcribed by the interviewer (JT) and shared back with the participant for verification. The eight key informants were individuals directly affected by cancer as patients or caregivers from underserved communities (i.e., Black, South Asian and East Asian, living in a rural and remote area, young adult diagnosed with cancer, francophone), with the exception of one informant who had other patient experience and extensive experience with patient engagement strategy development. Participant demographics are provided in Table 1. Fifty discrete pieces of information (suggestions, changes, and comments) were captured from the eight interviews.

The survey to provide feedback on the draft strategy was initially completed by 134 individuals, 90% who identified as white and 68% who identified their gender as women. Recognizing the homogeneity of the respondents, the Team decided more effort was needed to elicit the perspectives of a broader range of groups. For the second release of the survey, people affected by cancer from underserved communities were specifically sought. In total, 166 people completed the survey (from both rounds). The demographics of all those who provided feedback on all three strategy validation activities can be found in Table 1.

The data from the two waves of the survey were merged and frequency distributions of responses generated. The Team reviewed the aggregated responses to the survey but also decided that it was important to review the findings from respondents from underserved communities separately so as to identify and prioritize equity considerations being raised by underserved groups (such as black and racialized groups, young adults diagnosed with cancer, and those living in rural and remote areas). In total, 18 distinct additions and changes were suggested by survey respondents, 7 specifically by underserved groups. Importantly, 92% of all respondents (the same percentage when looking only at those from underserved groups) felt the strategy will help CCS reach the aim of partnering meaningfully and systematically with people in research funding (5% were unsure).

The CCS Advisory Council on Research provided feedback during a 20-min focus group at a virtual council retreat in January 2022. Council members were provided with the draft strategy in advance and open feedback was solicited. Seven distinct pieces of feedback were captured.

In total, the Team reviewed 76 pieces of feedback on the strategy. Grammar and minor changes (i.e., for understandability) were made. After removing duplicate feedback, the Team deliberated on the incorporation of the 36 pieces of distinct feedback into the draft strategy. All feedback was incorporated to the maximum extent possible, with only one piece of feedback from key informants not being acted on: an engagement activity around branding was kept in the appendix due to operational complexity and capacity. The Team met twice to review and incorporate changes and made significant additions and changes including: adding a vision statement (Advisory Council on Research feedback), modifying the aim, adding a guiding principle on safety (survey input), adding a patient engagement activity on trauma-informed resources and training (key informant input), modifying all sections of the strategy (multiple inputs), and moving activities from one level of importance to another based on feedback (multiple inputs). For instance, one activity (‘Connect the needs of researchers with the skills and interests of patient partners’) was moved from Important to Critical, one (‘Establish an Award for Excellence focused on patient engagement’) was moved from Critical to Important, and one (‘Design training for patients and researchers on patient engagement’) was moved from Important to High Priority (mid-level). The implication is that activities within the Critical level criteria will be implemented by CCS first, with the goal of resourcing and implementing these activities within one year. Activities were also modified based on feedback, such as being broadened (for example ‘Involve patients in supporting the development of research projects’ became ‘Engage patients in supporting the life cycle of research’), and additions were made to the evaluation section. In some cases, feedback was used to add detail to parts of the strategy. For example, feedback given in the survey included “Involving patients in establishing the distribution of research funds”, which was deemed to already be a part of the proposed governance and was included as a specific example in that section. It is operationalized by the engagement of patient members on our Advisory Council on Research, who help to guide our research funding directions, and in our expert review process where Patient/Survivor/Caregiver (PSC) reviewers participate. In both cases, patient advisory council members and PSC reviewers are equal members with the same level of authority and voting capability as other advisory council members and scientific reviewers. The strategy was approved by senior leadership at CCS for implementation in August 2022.

### Experience of strategy co-creation

**Fig. 2:**
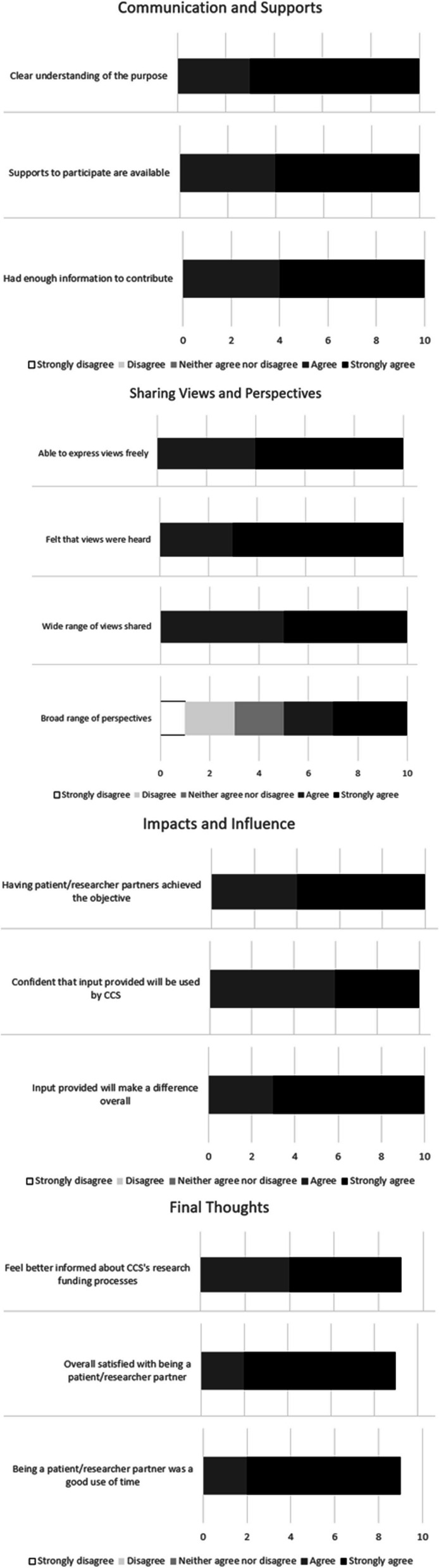
PPEET quantitative responses

Regarding process evaluation, 10 (of 14) participants completed the PPEET. Respondents rated all aspects of the process positively (communication and supports, sharing views and perspectives, impacts and influence, and final thoughts), with the exception of one question, “partners representing a broad range of perspectives”, where 5 respondents collectively were unsure, disagreed, or strongly disagreed with the statement. All quantitative responses can be seen in Fig. 2. To the 6 open-ended questions, 27 responses were received (3–6 responses for each). Positive feedback included acknowledgement of good facilitation and organization (n = 5), the creation of a safe space for honest feedback (n = 3), and the meaningful involvement and importance of involvement of patient partners (n = 10). Constructive feedback included the need to include more diverse perspectives (n = 6) and the need for continued tracking and dialogue on the outcomes of the work and next steps (n = 2). There was one comment on the need for funding to be secured for the initiative for several years to continue the work.

These responses to the PPEET highlight the collaborative nature of the Team and give examples of what worked well in the plan co-creation (i.e., facilitation, and patient partner inclusion). They also suggest improvements needed (i.e., diversity in perspectives) and future pain points to watch (i.e., continued tracking and dialogue).

## Discussion

The Strategy Development Team successfully co-created a multi-faceted patient engagement strategy for patient engagement in cancer research funding, specific to CCS. Lessons learned included: (1) the value of co-creation from the beginning, (2) the need for an action-oriented strategy, (3) the utility of facilitation techniques, and (4) the importance, and challenge of, designing for and incorporating equity when drafting the strategy. Co-creation as a Team was used from day one. While the staff lead (JT) created the terms of reference to describe the opportunity and expected commitment for potential Team members before Team members were approached, all other documentation and progress was made as a Team. This allowed the Team to take ownership as a whole of the project and the direction of the strategy. This was reflected in the PPEET results and anecdotal feedback, where Team members described feeling engaged and valued.

Team members prioritized an action-oriented strategy. For instance, the strategy includes specific patient engagement actions to undertake and assigns priority and a timeline for implementing the actions. This, coupled with the evaluation section, helps CCS to implement the strategy and report on outcomes. This level of detail also allows reproducibility.

In addition, facilitation techniques were used during workshops to co-design the strategy. Some facilitation techniques, such as ground rules for dialogue helped to set the virtual workshop space as a safe space for contribution, while other techniques such as Fist-to-Five consensus building helped ensure all Team members felt heard during deliberations and that if any Team member has reservations, they would not be missed. A modified version of Design Thinking and 10 × 10 idea creation was chosen for the workshops where patient engagement activities were being created and chosen. Design Thinking is an iterative, collaborative process meant to encourage creativity and efficiency—the creation of a product or solution with maximum value in a short timeframe. 10 × 10 idea creation is meant to support this with the creation of a large amount of ideas, or source material for potential patient engagement activities (the goal was 10 patient engagement activity ideas by each Team member in 10 min total) that could then be refined using Design Thinking. This was deemed successful as 60 distinct ideas for patient engagement activities were generated then refined into concrete actions in a single two-hour workshop, and the top 13 were prioritized and included in the finished strategy.

Finally, equity is an important and growing area of priority for both CCS and others, and the Team sought to apply an equity lens within the creation of the strategy. While the core Team is small (6 members), diversity was sought in both Team creation and in strategy validation. During strategy validation, underserved communities were approached specifically for feedback as key informants and survey respondents, and the data were analyzed by ‘centering at the margins’ [28]. The complete dataset was segmented by identity, and the feedback of those from underserved communities was examined. Importantly, the Team treated this feedback with greater importance than the rest of the dataset, meaning the Team strived to incorporate these changes first and foremost. This resulted in some of the changes and improvements to the strategy from a smaller, but valuable, portion of feedback. During the workshops the Team also discussed that during implementation of the strategy and all future work, an inclusive lens should be applied, and diversity and equity prioritized from day one, similar to co-creation.

While the creation of the strategy was successful and resulted in many lessons learned and positive outcomes, some limitations were noted. Most importantly, while diversity was valued by all Team members, there was a lack of diversity during strategy creation, and this was noted in the PPEET. The team could have been larger and more diverse; however, this was balanced by a need to be agile in the creation of the strategy. The team felt that diverse inclusion was a challenge primarily due to lack of relationships and partnerships with community at the time, as other facilitators to inclusion were in place—flexible hours for contribution and standard compensation rates ($25/hr) offered in low barrier ways. In addition, progress made in equity and diversity by CCS and others could improve approaches to equity and diversity now, but these relationships and knowledge were not present at the time of strategy initiation. More work is still needed to engage individuals from equity deserving groups so that they can believe that their feedback will be valued and reflected and worth the effort of providing it. CCS and other organizations are working and need to continue to work to find ways to earn the trust of groups that have been historically excluded or not brought into discussions. Other limitations include not knowing whether the strategy co-creation process described is reproducible or will generate buy-in and execution, and a lack of data on the impact of this strategy or patient engagement activities on research quality and research outcomes. While the strategy co-creation process has been well documented with detail for replication, this was a single case study and an organizational process for improvement. More examples of organizational patient engagement strategies in research funding, and their resulting outcomes, are needed.

Finally, a strategy that is not applied is not valuable, and so the Team sought a multi-faceted approach to mitigate this risk. (1) We incorporated a clear vision statement and aim, share these publicly and refer to them in practice; (2) We crafted the strategy to be action-oriented, including specific patient engagement activities that can be applied; (3) We included leadership throughout the development of the strategy. Our leadership (Executive Vice President, Vice President and Director) were invited to attend every workshop and were provided summary updates of progress every 3 months. This ongoing communication resulted in changes such as the addition of a section to the strategy called ‘Operational Considerations’, which informs both our implementation activities and leadership’s risk mitigation strategies and resourcing efforts; (4) We also sought specific leadership approval, so that the strategy could be given formal support for implementation and be included in operational plans ongoing on a year-by-year basis; (5) We launched communication across CCS and externally. This resulted in a strategy with strong leadership support and resourcing, as well as visibility and momentum from communication and promotion. Importantly, this strategy is being universally applied across CCS Research activities and is forming the basis of a cross-CCS Patient Engagement Framework outside of research activities. The strategy has changed CCS practices—for instance, our research competitions have changed based on the strategy. The strategy is also helping to inform other organizations’ policies and practices. While there is a lack of data on the effect of this strategy on research outcomes, at the time of submission CCS has initiated all 6 Critical level activities (with 5 being fully implemented and one in development). Four of the remaining 7 activities have also been initiated, and an initial process evaluation completed.

## Conclusion

A Team of patients, caregivers and researchers supported by CCS staff co-created a multi-faceted, action-oriented patient engagement in research strategy for cancer research funding. The strategy was validated in a 3-phase approach and approved by senior leadership for implementation. The experience of creating the strategy was noted as very positive overall, with a need for greater diversity highlighted. Lessons learned include the importance of using an equity lens throughout strategy creation, the value of co-creation from day one, the use of facilitation techniques, and in prioritizing actions to be included in the strategy. CCS is currently implementing the strategy and evaluating outcomes, as well as examining additional ways to meaningfully and systematically engage diverse voices in research and research funding. From the experience of creating and starting to implement the strategy, we can recommend a few specific actions to other research funders: (1) the use of co-creation from day one for a meaningful, relevant and credible strategy; (2) the inclusion of structured, facilitated meetings or workshops for developing the strategy—this helped to move the strategy forward even when other priorities competed for time and resources; and (3) the embedding of executable actions into the strategy—this allowed leadership to understand the resource allocation and needs of the strategy and discuss specific concerns, and ultimately, to support approval and implementation. This has also helped to communicate about the strategy both inside the organization and externally, as the action-oriented elements can easily be shared and understood. More on CCS’s patient engagement in research funding, including a summary of the strategy, is publicly available on our website at: www.cancer.ca/ENpatientengagement. Finally, there is an opportunity for future research on the patient engagement strategy and its effects on research outcomes, for more direct evidence on the effects of patient engagement on research quality.

### Supplementary Information

**Additional file 1**: GRIPP2 Long Form.

## Data Availability

The datasets used and/or analysed during the current study are available from the corresponding author on reasonable request.

## Abbreviations

CCS: Canadian Cancer Society
PPEET: Public and Patient Engagement Evaluation Tool
GRIPP2-LF: Guidance for Reporting Involvement of Patients and the Public-Long Form
