# Supplementary material for: Co-creation of a patient engagement strategy in cancer research funding
Source: Res Involv Engagem. 2023 Sep 29;9:86. doi: 10.1186/s40900-023-00501-x (PMC10542220; doi:10.1186/s40900-023-00501-x)
Supplement: Supplementary file 1 — Additional file 1: GRIPP2 Long Form. [file 40900_2023_501_MOESM1_ESM.docx]

**Supplemental File #1: GRIPP2 Long Form**

| **Section and topic** | **Item** | **Reported on page No** |
| --- | --- | --- |
| Section 1: Abstract of paper |  |  |
| 1a: Aim | Report the aim of the study | 1 |
| 1b: Methods | Describe the methods used by which patients and the public were involved | 1-2 |
| 1c: Results | Report the impacts and outcomes of PPI in the study | 2 |
| 1d: Conclusions | Summarise the main conclusions of the study | 2 |
| 1e: Keywords | Include PPI, “patient and public involvement,” or alternative terms as keywords | 2 |
| Section 2: Background to paper |  |  |
| 2a: Definition | Report the definition of PPI used in the study and how it links to comparable studies:  -The term patient engagement in our context refers to the inclusion of patients in research activities or in the funding process of research as contributors and/or decision makers | 3 |
| 2b: Theoretical underpinnings | Report the theoretical rationale and any theoretical influences relating to PPI in the study:  -Meaningful patient partnership within research funding is one of the integrated approaches for the success of CCS’ Research strategy  -Diverse backgrounds sought for Team participation including cancer experience, location, age  - Use of patient co-creation from outset (“nothing about us without us”) | 4 |
| 2c: Concepts and theory development | Report any conceptual models or influences used in the study:  -Used IAP2 Spectrum, Tamarack Institute ‘Creating Guiding Principles’, Design Thinking  -Influenced by: Tamarack Institute, McMaster University Collaborative for Health and Aging, Canadian Medical Association Patient Engagement Framework, CIHR SPOR Framework | 4-5 |
| Section 3: Aims of paper |  |  |
| 3: Aim | Report the aim of the study: -To: 1) co-create a patient engagement strategy in cancer research funding for the Canadian Cancer Society (CCS), 2) to describe the tools and approaches used in co-creation, 3) to describe successes and lessons learned | 3 |
| Section 4: Methods of paper |  |  |
| 4a: Design | Provide a clear description of methods by which patients and the public were involved:  -Four patients were approached and agreed to participate as core members of the Strategy Development Team  -8 Key informants and survey respondents were sought for validation | 4 |
| 4b: People Involved | Provide a description of patients, carers, and the public involved with the PPI activity in the study  -See Table 1 | 12-13 |
| 4c: Stages of Involvement | Report on how PPI is used at different stages of the study:  -To co-create strategy as a core member of the Team  -To validate strategy | 4, 5 |
| 4d: Level or nature of involvement | Report the level or nature of PPI used at various stages of the study:  -Co-creation as a member of the core Team  -Consultation as part of strategy validation | 4, 5 |
| Section 5: Capture or measurement of PPI impact |  |  |
| 5a: Qualitative evidence of impact | If applicable, report the methods used to qualitatively explore the impact of PPI in the study:  -Open-ended responses during validation from key informants, advisory council, and survey respondents  -Patient and Public Engagement Evaluation Tool | 5, 6 |
| 5b: Quantitative evidence of impact | If applicable, report the methods used to quantitatively measure or assess the impact of PPI:  -Survey responses during validation  -Patient and Public Engagement Evaluation Tool | 5, 6 |
| 5c: Robustness of measure | If applicable, report the rigour of the method used to capture or measure the impact of PPI:  -Patient and Public Engagement Evaluation tool validity and reliability stated | 6 |
| Section 6: Economic assessment |  |  |
| 6: Economic assessment | If applicable, report the method used for an economic assessment of PPI | N/A |
| Section 7: Study results |  |  |
| 7a: Outcomes of PPI | Report the results of PPI in the study, including both positive and negative outcomes  -Successful strategy co-development and validation including additional round of survey validation | 6-9 |
| 7b: Impacts of PPI | Report the positive and negative impacts that PPI has had on the research, the individuals involved (including patients and researchers), and wider impacts  -Lessons learned including challenges in equity, utility of facilitation, value of co-creation and action-oriented approach | 9-10 |
| 7c: Context of PPI | Report the influence of any contextual factors that enabled or hindered the process or impact of PPI  -Challenges with diversity, utility of facilitation techniques | 9-10 |
| 7d: Process of PPI | Report the influence of any process factors, that enabled or hindered the impact of PPI  -Leadership support (integrated approach for success of Research Strategy overall) | 4 |
| 7ei: Theory development | Report any conceptual or theoretical development in PPI that have emerged | N/A |
| 7eii: Theory development | Report testing of theoretical models, if any | N/A |
| 7f: Measurement | If applicable, report all aspects of instrument development and testing (eg, validity, reliability, feasibility, acceptability, responsiveness, interpretability, appropriateness, precision) | N/A |
| 7g: Economic assessment | Report any information on the costs or benefit of PPI | N/A |
| Section 8: Discussion and conclusions |  |  |
| 8a: Outcomes | Comment on how PPI influenced the study overall. Describe positive and negative effects  -Patient and Public Engagement Evaluation Tool results  -Multi-faceted, action-oriented strategy created | 8-9 |
| 8b: Impacts | Comment on the different impacts of PPI identified in this study and how they contribute to new knowledge  -Multi-faceted, action-oriented strategy created  -See lessons learned (co-creation, action-oriented approach, facilitation techniques) | 9-10 |
| 8c: Definition | Comment on the definition of PPI used (reported in the Background section) and whether or not you would suggest any changes | N/A |
| 8d: Theoretical underpinnings | Comment on any way your study adds to the theoretical development of PPI | N/A |
| 8e: Context | Comment on how context factors influenced PPI in the study  -See lessons learned (co-creation, action-oriented approach, facilitation techniques) and limitations (single case, reliability, diversity challenges) | 9-10 |
| 8f: Process | Comment on how process factors influenced PPI in the study  -See lessons learned (co-creation, action-oriented approach, facilitation techniques) and limitations (single case, reliability, diversity challenges) | 9-10 |
| 8g: Measurement and capture of PPI impact | If applicable, comment on how well PPI impact was evaluated or measured in the study  -Measured using validated survey, limitation of more (future) data on research outcomes needed | 8-9, 10 |
| 8h: Economic assessment | If applicable, discuss any aspects of the economic cost or benefit of PPI, particularly any suggestions for future economic modelling. | N/A |
| 8i: Reflections/critical perspective | Comment critically on the study, reflecting on the things that went well and those that did not, so that others can learn from this study  -See lessons learned (co-creation, action-oriented approach, facilitation techniques) and limitations (single case, reliability, diversity challenges) | 9-10 |
| PPI=patient and public involvement | | |
